# Post-mortem recrystallization of biogenic amorphous calcium carbonate guided by the inherited macromolecular framework

**DOI:** 10.1038/s41598-024-68037-y

**Published:** 2024-07-27

**Authors:** Jarosław Stolarski, Ismael Coronado, Marta Potocka, Katarzyna Janiszewska, Maciej Mazur, Alain Baronnet, Juncal A. Cruz, Olivier Grauby, Anders Meibom

**Affiliations:** 1 Institute of Paleobiology, Polish Academy of Sciences, Twarda 51/55, 00818 Warsaw, Poland; 2 Faculty of Biological and Environmental Sciences, University of Leon, Campus of Vegazana S/N, 24071 Leon, Spain; 3 Department of Antarctic Biology, Institute of Biochemistry and Biophysics, Polish Academy of Sciences, Pawińskiego 5a, 02106 Warsaw, Poland; 4 Department of Chemistry, University of Warsaw, Pasteura 1, 02093 Warsaw, Poland; 5 UMR 7325, CINaM, CNRS - Aix Marseille Université, 13288 Marseille, France; 6 Laboratory for Biological Geochemistry, School of Architecture, Civil and Environmental Engineering (ENAC), Ecole Polytechnique Fédérale de Lausanne (EPFL), Lausanne, Switzerland; 7 Center for Advanced Surface Analysis, Institute of Earth Sciences, Université de Lausanne, CH-1015 Lausanne, Switzerland

**Keywords:** Palaeontology, Geochemistry

## Abstract

In contrast to abiotically formed carbonates, biogenetic carbonates have been observed to be nanocomposite, organo-mineral structures, the basic build-blocks of which are particles of quasi-uniform size (10–100 nm) organized into complex higher-order hierarchical structures, typically with highly controlled crystal-axis alignments. Some of these characteristics serve as criteria for inferring a biological origin and the state of preservation of fossil carbonate materials, and to determine whether the biomineralization process was biologically induced or controlled. Here we show that a calcium storage structure formed by the American lobster, a gastrolith initially consisting of amorphous calcium carbonate (ACC) and amorphous calcium phosphate (ACP), post-mortem can crystallize into (thus secondary) calcite with structural properties strongly influenced by the inherited organic matrix. This secondary calcite meets many structural criteria for biominerals (thus called the biomorphic calcite), but differs in trace element distributions (e.g., P and Mg). Such observations refine the capability to determine whether a fossil carbonates can be attributed to biogenic processes, with implications for the record of life on Earth and other terrestrial planets.

## Introduction

The capability to distinguish between biogenic and abiogenic mineral structures, especially at microscopic length-scales, is a fundamental challenge for paleontologists, geologists, geochemists, and astrobiologists in their attempts to unravel the history of life on Earth and other rocky planets^1^. Reliable criteria for a biogenic origin of mineral structures are difficult to establish (such as endogenous organic content or specific crystal organization) and often appear ambiguous under in-depth scrutiny^2,3^. Nevertheless, attempts have been made to define features of biogenic minerals (such as carbonates, phosphates, and silica) that distinguish them from abiotic crystals^4–7^. For example, biogenic crystals typically exhibit nanocomposite, organo-mineral structures consisting of sub-particles of with quasi-uniform size distribution, controlled aggregation and texture, with high levels of spatial organization and preferential crystallographic orientation—all organized into higher-order hierarchical structures^8^ typically exhibiting complex macromorphologies. Such distinct features of biominerals are thought to be the result of biologically orchestrated mineralization processes in which organic macromolecules (often referred to as the organic matrix) interact with inorganic ions within confined mineralization compartments^9,10^.

It has recently been recognized that the skeletons, shells, tests, etc. of many groups of calcifiers are formed through the crystallization of originally poorly ordered carbonate precursors, such as amorphous calcium carbonate (ACC)^11–13^. The biological control of this process is still poorly understood but evidence suggests that intracellular, nanometer-size and disordered precursors are transported to calcification sites, where they aggregate and form crystals by particle attachment crystallization^14–18^. The disordered carbonate phase may crystallize at the calcification site within a few hours but can also remain disordered on much longer timescales, stabilized by organic macromolecules, phosphates, silica, and Mg^2+^ ions at high supersaturation levels^19–24^. This emerging paradigm regarding biocrystallization from amorphous precursors, alongside poorly understood endogenous and exogenous factors affecting the crystallization process, prompts several inquiries concerning the earliest diagenetic processes in incompletely crystallized biomineral structures. What impact does endogenous organic matter within amorphous precursors have on the structural characteristics of resulting biocrystals? Can organic matter involved in biomineralization maintain its functional influence during the formation of secondary structures arising from processes such as dissolution and re-precipitation of secondary mineral phases? Many of these processes are challenging to visualize due to the very thin zone of amorphous precursor crystallization.

Here, we explored some structural aspects of crystallization of disordered biogenic carbonate in objects large enough to allow macro-, micro-, and nano-scale observations to be made. The objects under consideration are gastroliths, which are large (up to a few centimeters in diameter) dynamic calcium storage structures formed in the stomachs of lobsters (crustacean arthropods) and composed primarily of amorphous calcium carbonate^25–27^. When lobsters grow, these gastroliths serve as reservoirs of calcium ions for calcification of their cuticles at the end of the molting cycle (ecdysis). Under natural physiological conditions (i.e., inside the lobster body) the amorphous carbonate material is assimilated in the stomach by the organism immediately during molting and does not crystallize^28^. If extracted and preserved under dry conditions, gastroliths will also preserve their amorphous state, but crystallization is initiated if the humidity increases and water absorption takes place^29,30^. In our experiments, the crystallization of initially amorphous gastroliths was controlled by controlling hydration. The only biogenic influence on the crystallization process was the inherent macromolecular network of organic matrix embedded in the gastrolith structure and its original chemical variations. The characterization of the resulting crystalline calcium carbonate provided detailed information about the extent to which specific structural and/or chemical properties of a fossil crystalline carbonate can be ascribed to biological processes.

## Materials

**Figure 1:**
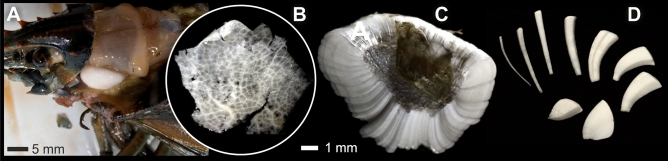
Gastrolith morphology. (**A**) Well-developed gastrolith in dissected American lobster (*Homarus americanus*). (**B**) The cardiac stomach wall in discoid areas of the monolayered epithelium where the growth of gastrolith columnar units take place; calcification occurs in polygonal compartments (here empty; transmitted light). (**C**) Longitudinally broken mature gastrolith to show arrangement of columnar units. (**D**) Diverse shapes of columnar units after bleaching and natural disassembly of the gastrolith. ZPAL V.31/14.

**Figure 2:**
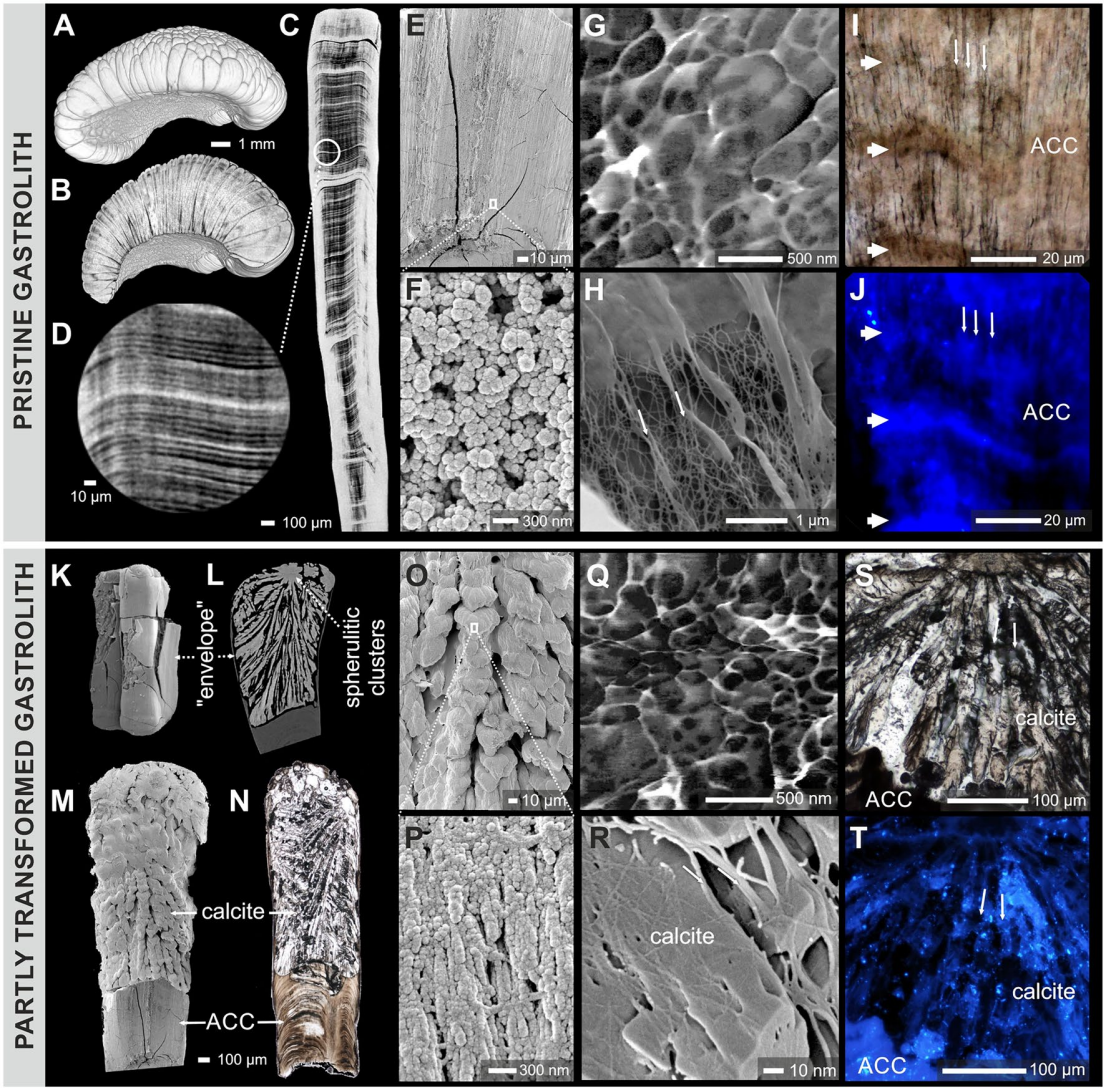
Hierarchical structure of original amorphous mineral phases of lobster gastrolith vs. biomorphic calcite. (**A**–**D**) Mature and pristine gastroliths showed composite, columnar organization on the outer surface (**A**), columnar units in virtual sections (**B**) and, under higher magnification, fine-scaled banding with each band ca. 5 µm wide (**C**,**D**). The amorphous gastrolith region was composed of nanograins ca. 100–200 nm in diameter (**E**–**G**). Within amorphous regions, dense organic fibers are exposed by selective mineral etching (**H**). These fibers are visible as longitudinal (thin arrows) but also horizontal structures (thick arrows) in transmitted light (**I**) and in epifluorescence, after calcofluor-white staining of chitin (**J**). After experimental, partial hydration the columnar units typically consisted of three regions: an unaltered lower part, a transformed upper part, and a skin-like envelope (**K**–**N**). The biomorphic calcite typically showed braid-like micro-scale organization of rhombohedral units (**O**) of nanogranular texture (**P**,**Q**); see also Fig. 3J,K. The biomorphic calcite structures were also associated with organic fibers that included chitin ((**S**,**T**); calcofluor-white staining). A–D and L: X-ray microcomputed tomography visualization; E, F, H, K, M, O, P, and R: FESEM; I. N, and S: optical transmitted microscopy; J. and T: epifluorescence microscopy; G and Q: atomic force microscopy (phase images).

About 30 adult male hard-shell American lobsters (*Homarus americanus*) from the Gulf of Maine, Tenant Harbor area (St. George, ME, USA) were imaged with ultrasound using a portable ultrasound machine to determine gastrolith development; only lobsters with fully developed, i.e., pre-molt stage, gastroliths were dissected and the gastroliths preserved in 99.5% analytical grade ethanol (Fig. 1). Prior to the transformation experiments, one such gastrolith, maintained at room temperature and under dry air conditions to prevent crystallization^31^, was subjected to high-resolution 3D tomography scanning to reveal the hierarchical structure of the original amorphous mineral phases (Fig. 2). A complete gastrolith consists of more than one hundred individual columnar units, which were mechanically extracted for preparation of thin sections or for use in crystallographic and geochemical analyses. The material is housed at the Institute of Paleobiology, Polish Academy of Sciences, Warsaw (abbreviation ZPAL).

In the transformation experiment, the gastrolith was immersed into a mixture of 99.5% analytical grade ethanol and analytical grade water to modulate the water content in the bulk medium. The high volume ethanol/water ratio (9:1) ensured that the dominant mineral phase formed through the transformation of amorphous calcium carbonate was calcite^32^. All experiments were carried out at room temperature. The gastrolith was retrieved from the ethanol/water mixture after 30 days, rinsed with isopropanol, and kept dry until further analysis. The extracted columnar units were embedded in cold epoxy resin before thin sectioning. Block-face cuts and thin sections were polished with alumina and colloidal silica using ethylene glycol and suspensions free of water. For XRD analyses, the pristine and experimentally altered gastrolith columnar units were gently ground in an agate pestle and mortar.

A diverse set of analytical tools were used to compare structural and geochemical features of modern and fossil samples. Details of optical and epifluorescence microscopy, micro-CT, high-resolution transmission electron microscopy (HR-TEM), field emission scanning electron microscopy (FESEM), atomic force microscopy (AFM), Raman spectroscopy, high-resolution X-ray powder diffraction (XRD), electron backscatter diffraction (EBSD), and electron microprobe analysis (EMPA) are provided in the Supplementary Information.

## Results and discussion

### Structure and mineral phases

#### The pristine gastroliths

**Figure 3:**
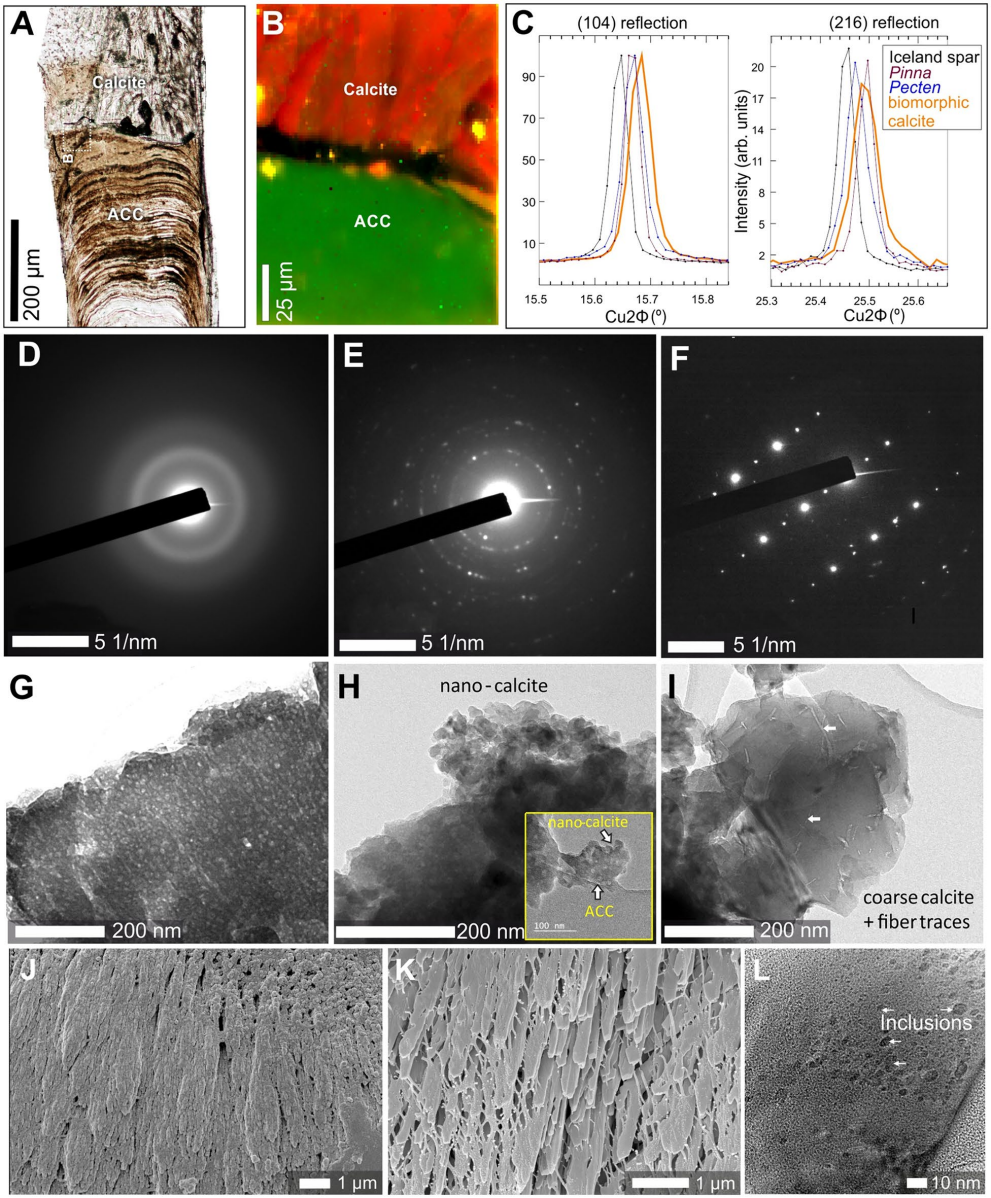
Mineralogic and selected crystallographic features of pristine amorphous phases and secondary biomorphic calcite. Transmitted optical light image of thin-sectioned gastrolith columnar unit (**A**) with a distinct border between amorphous (bottom) and crystalline (top) regions. (**B**) Micro-Raman map of pristine (bottom, composed of disordered carbonate, ACC) and altered (top, composed of calcite) gastrolith regions. (**C**) Angular positions of selected X-ray diffraction reflections (104, left plot) and (216, right plot) in biogenic (*Pinna* and *Pecten* bivalve shells), abiotic Iceland spar calcite, and gastrolith biomorphic calcite. Note the characteristic shifts between abiotic Iceland Spar and biogenic/biomorphic calcites to higher reflection angles and the broadening of the peak in biomorphic calcite in comparison with other samples. (**D**–**F**) Selected area electron diffraction (SAED) patterns and corresponding bright-field TEM images of ACC (**G**–**I**). Note the diffuse halo in D, characteristic of material with no long-range order, i.e., amorphous, absorption contrast without diffraction contrast (**G**); nanocrystalline biomorphic calcite + ACC (**E**,**H**), and fully monocrystalline calcite (**F**,**I**). The same selected aperture is maintained from D to F. Crystalline calcite fibers at the surface (**J**) and in fractured braid-like unit (**K**); note organic framework and rhombohedral faces at the endings of fibers ((**J**,**K**), SEM). (**L**) Numerous inclusions within calcite fibers may represent organic macromolecules that were originally involved in biomineralization process (TEM). ZPAL V.31/14.

**Figure 4:**
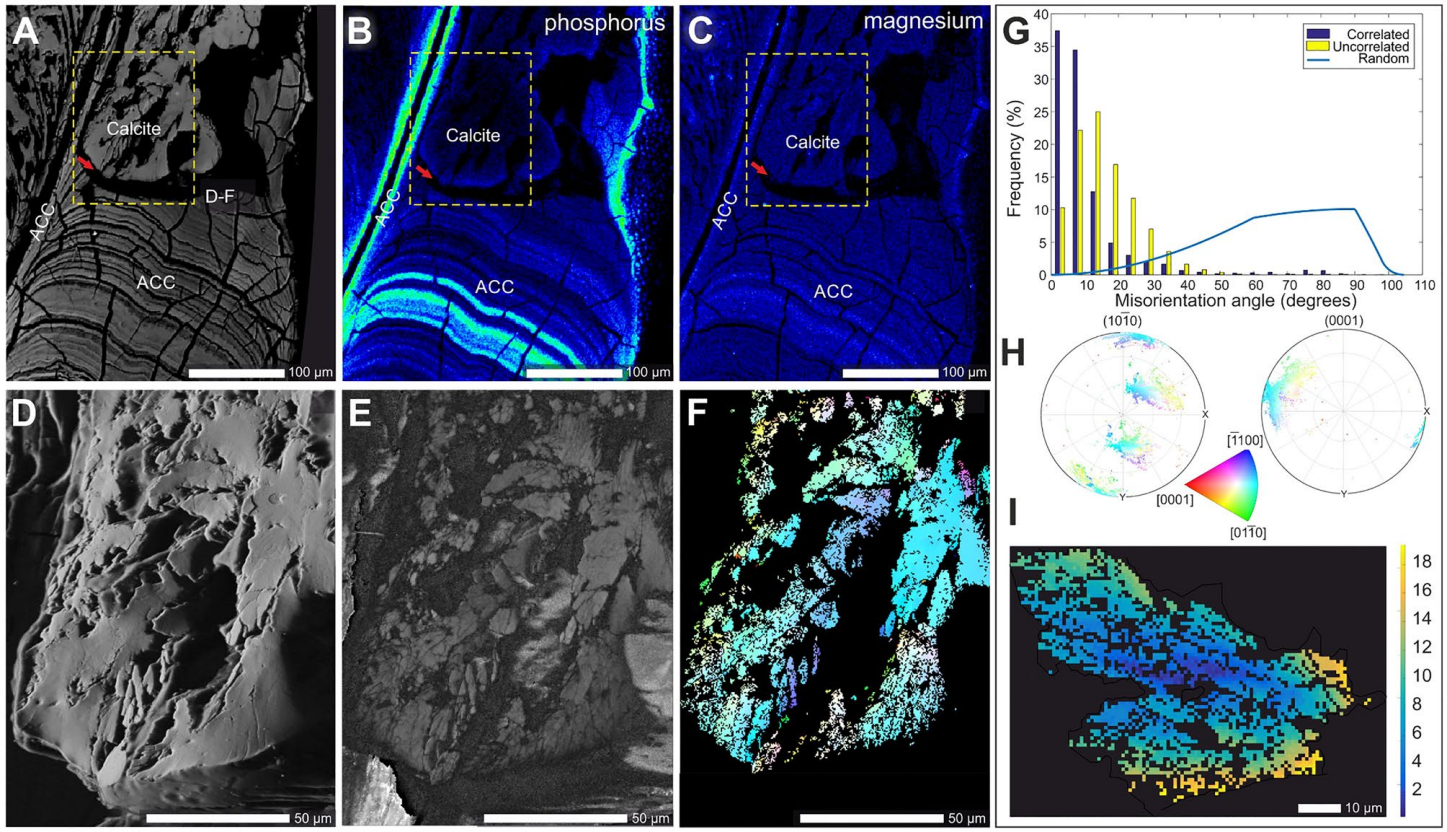
Crystallographic and selected geochemical features of pristine amorphous phases and secondary biomorphic calcite. (**A**–**I**) Typical gastrolith columnar unit with sharp boundary at the dissolution–precipitation front (indicated by red arrows in (**A**–**C**)) between amorphous phases (ACC) and secondary crystalline calcite (braid-like structures). The pristine amorphous minerals show strongly heterogenous (banded) distribution of P correlated with weaker variations in Mg ((**B**,**C**) see also Supplementary-Fig. 3). These trace elements are homogenously distributed in lower concentrations in the interior of the crystallized gastrolith structure, except along the edge, which is enriched in Mg and P. SEM micrograph of sectioned, braid-like structure (**D**) directly compared with band-contrast image (**E**) which shows morphology and grain size compared directly with EBSD orientation maps. The correlated and uncorrelated misorientations of biomorphic calcite crystals show very low angles (**G**), pointing to high crystallographic control of all units; histograms show correlated (blue bars) and uncorrelated (yellow bars) misorientation of crystals; a random crystal orientation distribution is computed for this crystal symmetry and indicated with the blue line; pole figures (**H**) indicate the crystallographic orientation of calcite crystals with reference to the planes (10 $\overline{1}$ 0) and (0001), crystallographic axes indicated by the color code; misorientation images (vertical scale in degrees) of calcite crystals (**I**). (**A**) BSE image; (**B**,**C**) EMPA elemental maps; (**D**) SEM images; (**E**) band contrast image, and (**F**,**I**) EBSD orientation maps. ZPAL V.31/14.

Macroscopically, the mature and pristine gastroliths of *Homarus americanus* were composed of more than hundred, tightly packed columnar units of diverse shapes and sizes that adhere to polygonal compartments of the cardiac stomach wall (Figs. 1, 2A,B). In longitudinal cuts, the pristine gastrolith columnar units showed fine-scale transverse banding (bands ca. 5 µm wide; Fig. 2C,D) and dense longitudinal striation (Fig. 2E,I). Using FESEM and AFM, it was observed that the pristine gastroliths exhibited the classical nanogranular structures with subunits ca. 100–200 nm in diameter (Fig. 2F,G). These nanograins were also distinct in FIB extracted lamellae of pristine gastrolith observed by TEM (Supplementary Fig. 1A–D). Etching of the mineral phase of pristine gastroliths revealed dense, banded concentration of longitudinally oriented organic fibers whose chitinous composition was demonstrated by calcofluor-white staining (Fig. 1H,J). Other studies have shown that functionally similar gastroliths in crayfish have organic matrix consisting of proteins, polysaccharides (proteoglycans), and lipids^33^. Micro-Raman maps (Fig. 3A,B; Supplementary Fig. 2) and selected area electron diffraction (SAED) patterns (Fig. 3D) indicated that pristine gastroliths were composed primarily of disordered/amorphous calcium carbonate (ACC), with a minor component of disordered/amorphous calcium phosphate (ACP) (Supplementary Fig. 2). The pristine gastrolith mineral phase exhibited a banded distribution of the trace elements P and Mg, with P- and Mg-enriched bands observed mainly in the lower portion of pristine gastrolith columnar unit (Fig. 4A–C; Supplementary Fig. 3). Electron microprobe maps indicated high co-variation between the P/Ca and Mg/Ca (Pearson correlation index between metal/Ca ratios were 0.74 and 0.86, respectively; Supplementary Fig. 3).

#### The biomorphic calcite

After controlled hydration, all examined gastrolith columnar units (ca. 100) subjected to experimental treatment consisted of two parts, separated by a distinct porous interface (ca. 10 µm wide): an unaltered lower part and transformed upper part (Fig. 2L–N), as well as a mineral envelope or ‘skin’ surrounding the columnar unit (Fig. 2K,L). Micro-Raman maps, high-resolution XRD, selected area electron diffraction (SAED) patterns, and EBSD phase images indicated that transformed, crystalline regions of experimentally treated gastrolith columnar units were composed almost entirely of calcite (Fig. 3B,F,I; Supplementary Figs. 2, 4). However, within this crystalline material some disordered carbonate material could still be detected embedded between calcite crystals (e.g. Fig. 2E,H; Supplementary Figs. 5, 6). No peaks characteristic to calcium phosphate (hydroxyapatite) was detected (Supplementary Fig. 5).

The phase transformation was initiated in the upper parts of the columnar units, where the last formed (i.e., end of pre-molt stage) and most fragile parts of the lobster gastrolith exist^33,34^. The first formed secondary structures were spherulitic clusters (Fig. 2L, Supplementary Fig. 4). Once initiated, the transformation process seems to have progressed as a dissolution-reprecipitation front with a sharp boundary between the chemically banded, ACC structure and the braid-like calcite (Figs. 2L–N, 3A,B, 4A–C). Due to the hierarchical organization of these secondary calcitic structures, which morphologically resemble some biologically-controlled calcium carbonate structures (e.g., shingles in *Acropora* scleractinian corals^35^), the braid-like organization of calcitic structures is herein called biomorphic.

The ACC-calcite transformation cause a significant reduction in molar volume and increase in density of the end product, resulting in significant porosity^36,37^. The measured average porosity of experimentally treated gastroliths was ~ 34.4% (vs. ~ 31.8 porosity calculated in abiotic ACC-calcite transformation), with the highest porosity in the upper parts of columnar units (Supplementary Fig. 7); this suggests that the dissolution-reprecipitation process resulted in a loss of soluble organic macromolecules along with some elements from the original mineral phase. The mobilization of elements from the original amorphous phase is supported by the observed homogenous distribution of Mg and P in the secondary calcite with an average concentration of Mg is 2566 ppm and 2341 ppm of P, in contrast to the banded distribution of Mg and P in pristine gastrolith, with an average amount concentration of Mg is 6987 ppm and 27,113 ppm of P (Fig. 3B,C).

The crystalline calcite upper parts (Fig. 2L–O) formed a typical biomorphic shape with an elaborate braid-like structure which crystallized from the edge of the columnar unit in spherulitic clusters with a radiating structure (Fig. 2L; Supplementary Fig. 5). These braid-like structures were composed of stacks of calcite units (ca. 20 µm in length, Fig. 2O) that, at higher magnification, exhibited nanogranular-fibrous structure (nanograins ca. 100–200 nm in diameter aligned into fibers; Fig. 2P,Q,J). The calcite fibers often show rhombohedral faces at the ends of fibers (Fig. 3K, see also rhombohedron habits of calcite crystals in interphase and porous areas in Supplementary Fig. 1I, J).

The calcite crystals were associated with an organic matrix that included chitin (Fig. 2R–T; calcofluor-white staining, see also Fig. 3K). Observed by TEM, the calcite also revealed the presence of nanometer-scale intracrystalline inclusions with low electron density (most likely organic; Fig 3I,L; Supplementary Fig. 1H). The selected (104 and 216) diffraction reflections of the biomorphic calcite (Fig. 3C) were shifted to higher angles in both reflections (Supplementary Table 1), in a manner similar to biogenic calcites (*Pinna*, *Pecten* bivalve shells), but distinctly different from abiotic calcite, e.g., Iceland spar. Relative to Iceland spar, substantial spectral broadening (up to 1.2%) was also observed in biomorphic calcite crystals, as is the case for other biogenic calcites^38^.

Crystal misorientation analysis based on EBSD maps provide insights into the degree of crystallographic^39^ and biomineralization control^40,41^. Briefly, the nearly random distributions of misorientation axes indicate that individual crystals are not ordered with respect to each other indicating a low degree of control exerted by an organism on its mineralized structures. Conversely, strictly constrained dispersion of misorientations indicate that individual crystals are well ordered with respect to each other as in well-known examples of biologically controlled biomineral structures such as bivalve nacre^41^ or subunits of sea urchin teeth^42^.

Applied to the first formed spherulitic clusters and later formed, braid-like units within the gastroliths, an interesting pattern is observed. Uncorrelated crystal misorientation between the spherulitic clusters exhibited large angles, suggesting relatively low crystallographic control during the earliest phase of crystallization (Supplementary Fig. 5D). However, the correlated crystal misorientation within the spherulitic clusters (Supplementary Fig. 4D) and within braid-like units i.e., closer to the dissolution-coprecipitation front (Fig. 4G; Supplementary Fig. 6E), had very low angles, akin to mineral products resulting from a biologically tightly controlled process.

### The transformation process: a model

These experimentally transformed gastrolith columnar units preserving parts of their original ACC along with the secondary crystalline calcite phase provide important insights into the mechanisms of phase transformation. Only the upper portions of the gastrolith columnar units, characterized by initially lower levels of Mg and P, underwent crystallization (2056 ppm and 1811 ppm respectively). In contrast, the lower sections of the columnar units and their mineral envelopes retained the original disordered mineral phase. These regions, where the disordered mineral phase persisted, exhibited elevated concentrations of Mg and P ((17,351 ppm and 58,161 ppm respectively, see Supplementary Fig. 3), which are known to stabilize amorphous phases^19,22,43,44^. However, the Mg content in ACC is correlated with its solubility^45^, while the stability of ACC can also be influenced by organic additives^46^. Further experiments could provide insight into whether prolonged transformation times would facilitate the phase transformation of the amorphous regions described in this experiment.

**Figure 5:**
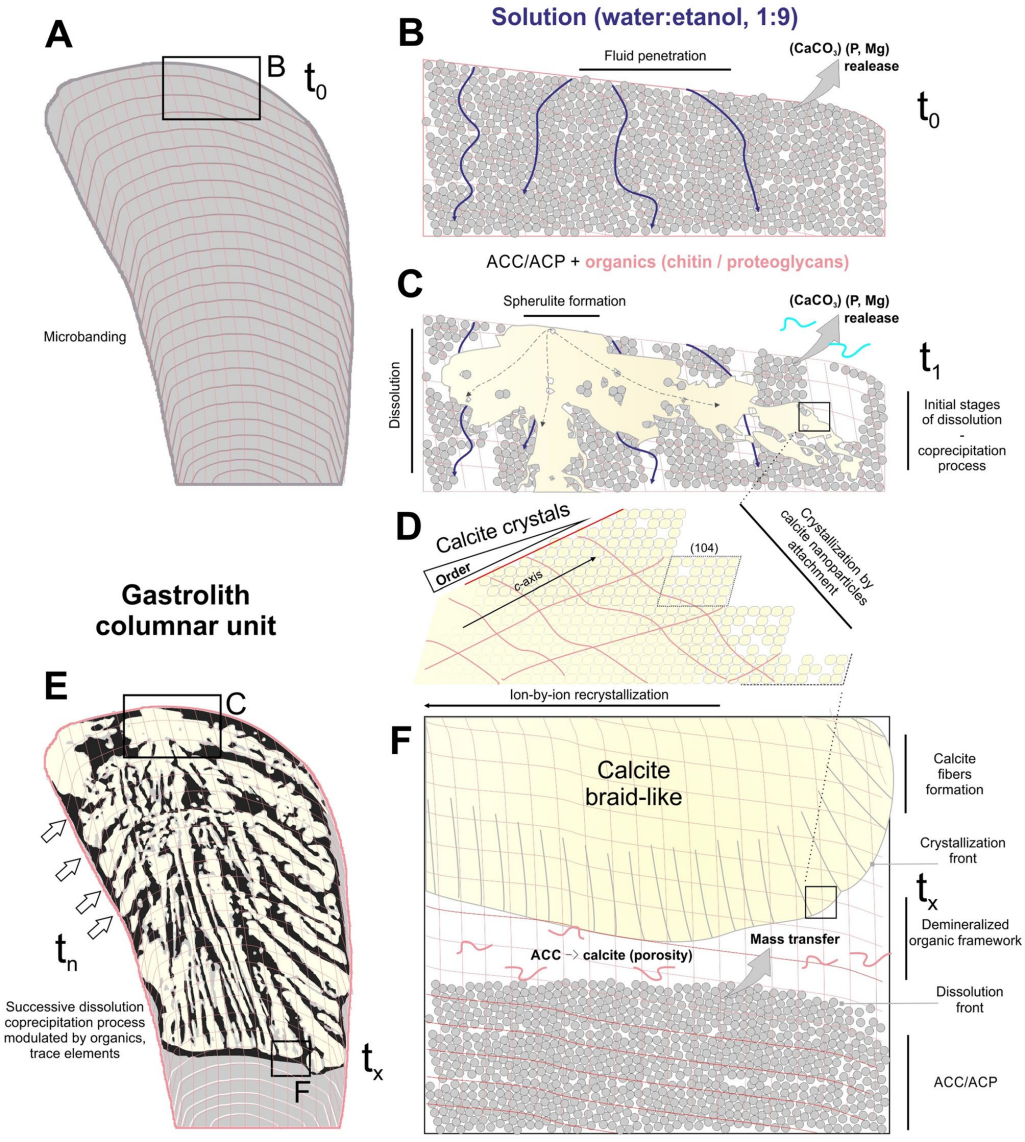
Proposed model of experimental mineral phase transformation of gastroliths in water–ethanol solutions. Transformation starts at time t_0_ (**A**,**B**) with penetration of the water–ethanol fluid through the distal point (i.e. the last formed and most fragile part of the columnar unit) and successive dissolution of the pristine biomineral components (ACC/ACP nanograins associated with organic chitin/proteoglycan). Dissolution of pristine biomineral and coprecipitation of secondary mineral phase starts with spherulitic clusters formation ((**C**), time t_1_). The secondary calcite nanoparticles aggregate within organic framework and form calcite fibrous structures that eventually merge through an ion-by-ion exchange to form mature braid-like structures (**D**,**F**). The formation of braid-like units is modulated (t_n_) and spatially constrained by the banded distributions of organic matter and trace elements concentrations (**E**).

For the observed changes in composition and structure of pristine gastroliths, we propose a sequence of events as discussed in the following and summarized in Fig. 5.

Transformation starts at time t_0_ (Fig. 5A,B) with penetration of the water–ethanol fluid through distal, i.e., into the last formed and most fragile part of the columnar unit, and progression of dissolution of the pristine amorphous components, i.e., ACC/ACP nanograins, was guided/constrained by the organic (chitin/proteoglycan) framework.

Dissolution of pristine biomineral and coprecipitation of secondary mineral phase initially resulted in the formation of spherulitic clusters (time t_1_; Fig. 5C): After the dissolution of the original ACC/ACP structure, partially disordered calcite nanoparticles were precipitated along the spongy, organic framework exposed by the dissolution front (a similar organic framework has been observed in experimentally demineralized gastroliths of red claw lobsters^47,48^). These calcite nanoparticles, acted as seeds of crystallization by particle attachment^15^, and subsequent crystal growth led to the formation of calcite fibrous structures (Figs. 2P, 3J). These fibrous calcite structures exhibited the classical nanogranularity, remnants of the original chemical banding, and contained inclusions of most likely organic framework (Figs. 2P,R, 3J,L). The crystalline calcite fibrous structures eventually merged together through continued growth (Fig. 5D), conceivably through an ion-by-ion exchange that often leads to the characteristic rhombohedral faces, as observed their endings (105° angles between (104) faces, Fig. 3K; Supplementary Fig. 1J; a similar process of recrystallization of calcitic granules has been observed in earthworms^49^.

The dissolution-reprecipitation process was most likely modulated and spatially constrained by the banded distributions of organic matter (chitin fibers) and trace elements concentrations (Fig. 2I,J; 4B,C). During the progressing dissolution-reprecipitation process, the transformation was constrained and perhaps temporarily halted by this banding. This process was repeated several times (time t_n_; Fig. 4E), which would explain the formation of stepped, braid-like structures composed of crystallographic aligned stacks of calcite units (ca. 20 µm in length, Fig. 2O). The *c*-axis of calcite fibers and microscale calcite units were oriented parallel to the morphological long-axis and remnants of original ACC were detected only at the growing front of calcite crystals (edges and within the "lobes" of crystal bundles; Fig. 4D–F,H,I; Supplementary Fig. 5).

### Biomineral zombies: broader impacts and conclusions

Some of the most ambiguous and inconsistent terms used to describe the state of preservation of fossilized structures of biological origin are "excellent" and "pristine". Most commonly, excellent preservation means that the fossil remains show macro- or microstructural features that appear visually unaltered. It may also refer to the preservation of mineralogy that is considered primary or refer to the preservation of biogeochemical and isotopic signatures whose composition and spatial distribution is considered preserved intact within the biomineral. The latter is essential for reliable biogeochemical proxy-based paleoclimate and paleophysiological reconstructions^10,35,36^. To use fossil remains as such proxies, various tests have been developed to ensure that only the best-preserved samples are selected. For example, fossil coral skeletons that, for the vast majority, are composed of aragonite (a metastable variety of calcium carbonate) are considered well-preserved if they meet the structural (overall morphology, microstructural patterns), mineralogical (occurrence of only aragonite mineral phase), geochemical, and isotope criteria of non-diagenetically altered samples^50^. In many cases, less rigorous tests are used to determine the state of preservation of biogenic structures consisting of more stable varieties of calcium carbonate (e.g., low-magnesium calcite), based on the most common assumption that the preservation of fine structural features is a sufficient to establish highly pristine preservation^51^. However, experimental studies have demonstrated that, e.g., foraminifera tests may undergo significant oxygen isotope exchange with fluids without showing any discernible textural changes^52–54^. Consequently, even "excellently" preserved (i.e., optically translucent, or “glassy”) fossil foraminifera tests may in fact be substantially altered with respect to their isotopic compositions.

While processes involving the transformation of ACC phases into crystalline structures have been extensively studied^55,56^, our work is specifically focused on the relatively less investigated transformation of biogenic ACC, as its occurrence is typically confined to very narrow, nano/micrometric zone within newly formed skeletons. It is commonly acknowledged that in many groups of organisms an amorphous precursor is the starting point of biomineral formation, and after period of stabilization by organic macromolecules and/or inorganic components (e.g., P or Mg) this amorphous phase transforms into a crystalline structure^11,13,17,18,57,58^. The resulting crystalline structures—generally considered primary—may show various structural and/or biogeochemical alteration in comparison to the originally formed amorphous precursors. Biomineral structures that undergo diagenetic transformation, but apparently look like primary ones, we propose to call biomineral zombies (from the apparent life of the dead according to voodoo believers). The transformation of originally amorphous gastroliths serves as a powerful example and model of such alteration.

In the gastroliths studied here, the biomorphic calcite crystalline structures developed within a pre-existing (primary) biogenic macromolecular framework, exhibited many structural properties that would be considered pristine for biominerals, i.e., high levels of spatial organization, complex morphologies, bi-composite nature, preferential crystallographic orientation, and crystal lattice parameters typical of biogenic minerals. However, at the same time, this biomorphic calcite lacked features that are typically ascribed to pristine biominerals, such as the banded, heterogenous distribution of trace elements (likely resulting from physiologically driven fluctuations in the supply of ions to the calcification site) and a nanogranular organization comparable to the original amorphous gastrolith (Fig. 2F). The results of our experimental crystallization of biogenic amorphous phase therefore point out that the amount and spatial distribution of amorphous precursors, the spatial organization of macromolecular framework that influence calcium carbonate crystallization^59^, the geochemical composition of the precursor ACC and associated organic phases, as well as post-depositional exposure of the pristine biomineral to the external environment, impose a range of structural and compositional characteristics on the resulting crystalline phase that are not present in originally deposited, amorphous biomineral. Therefore, interpretations of, e.g., the nanoparticulate fabric found in phosphatized Ediacaran and Cambrian fossils as evidence of original crystallization by particle attachment of ACC particles^1^ are uncertain, because they could also reflect the nanoscale effects of a secondary phosphatization process^60^. Moreover, partly or entirely amorphous biomineral structures of biological samples preserved in water solutions of ethanol or formaldehyde, despite their overall good preservation of the tissue, can be entirely transformed with structures representing "biomineral zombies".

Better understanding the complexity of physico-chemical phenomena that accompany biomineral transformation since the very first moments after deposition is essential to improve the use of fossil biominerals as paleoenvironmental proxies and interpret the geological record during the Phanerozoic in general.

### Supplementary Information

Supplementary Information.

## Data Availability

Data generated or analysed during this study are included in this published article and its supplementary information files.
