# Supplementary material for: Post-mortem recrystallization of biogenic amorphous calcium carbonate guided by the inherited macromolecular framework
Source: Sci Rep. 2024 Jul 27;14:17304. doi: 10.1038/s41598-024-68037-y (PMC11283521; doi:10.1038/s41598-024-68037-y)
Supplement: Supplementary file 1 — Supplementary Information. [file 41598_2024_68037_MOESM1_ESM.docx]

Supplementary Information for

Post-mortem recrystallization of biogenic amorphous calcium carbonate guided by the inherited macromolecular framework

**Jarosław Stolarski^1^, Ismael Coronado^2^, Marta Potocka^3^, Katarzyna Janiszewska^1^, Maciej Mazur^4^, Alain Baronnet^5^, Juncal A. Cruz^2^, Olivier Grauby^5^, Anders Meibom^6,7^**

^1^*Institute of Paleobiology, Polish Academy of Sciences, Twarda 51/55, PL-00-818 Warsaw, Poland;*

^2^*University of Leon, Faculty of Biological and Environmental Sciences, Campus of Vegazana s/n, 24171 Leon, Spain;*

^3^*Department of Antarctic Biology, Institute of Biochemistry and Biophysics, Polish Academy of Sciences, Pawińskiego 5a, PL-02-106 Warsaw, Poland;*

^4^*Department of Chemistry, University of Warsaw, Pasteura 1, PL-02-093 Warsaw, Poland;*

^5^*CINaM - UMR 7325, CNRS - Aix Marseille Université, 13288 Marseille, France*

*^6^Laboratory for Biological Geochemistry, School of Architecture, Civil and Environmental Engineering (ENAC), Ecole Polytechnique Fédérale de Lausanne (EPFL)*

*^7^Center for Advanced Surface Analysis, Institute of Earth Sciences, Université de Lausanne, CH-1015 Lausanne, Switzerland.*

Jarosław Stolarski (corresponding author). Email: stolacy@twarda.pan.pl

**Methods**

**Optical microscopy**. Polished sections of gastrolith columnar units were examined using a Nikon Eclipse 80i transmitted light microscope fitted with a DS-5Mc cooled camera head. Observations were conducted in transmitted and polarized light.

**Raman microscope**. The Raman measurements were performed with LabRAM

800 HR confocal microscope (Horiba Jobin Yvon) equipped with a diode-pumped

Nd:YAG laser (Spectra-Physics) operating at 532.3 nm (ca. 2 mW power on the sample). The individual spectra were recorded using 1,800 groove/mm holographic grating, while for the acquisition of maps, the 600-groove/mm grating was used. The most convenient signals allowing for identification of the calcite and aragonite polymorphs are grouped in the 100 cm^−1^ to 300 cm^−1^ regions. These peaks, associated with lattice vibrations, appear at 205 cm^−1^ and 153 cm^−1^ for aragonite and at 281 cm^−1^ and 153 cm^−1^ for calcite. The analysis of the maps was performed employing the modeling option of the Labspec software (Horiba Jobin Yvon).

**Scanning Electron Microscopy (SEM)**. Polished sections of gastrolith units were lightly etched in Mutvei’s solution following described procedures^1^ and then rinsed with Milli-Q water and air-dried. After drying, the specimens were put on stubs with double-sticking tape and sputter-coated with conductive platinum film. Analyses were made using a Phillips XL20 scanning electron microscope at the Institute of Paleobiology, Warsaw, Poland.

**X-ray microtomography**. 3D visualizations of the internal structure of the gastroliths and their columnar units were made with Zeiss XRadia MicroXCT-200 system (referred to as Micro-CT). Scans were performed using the following parameters: voltage: 20 kV, power: 10 W, exposure time: 20 s, pixel size: 1.08 μm, 1601 projections. Three-dimensional images were obtained by processing with the AVIZO7.1 Fire Edition software. All micro-morphological and micro-structural analyses were performed at the Institute of Paleobiology, Polish Academy of Sciences.

**Atomic Force Microscopy (AFM).** The AFM allows for the determination of the 3D surface topology of the biomineral at nanometer resolution. Following established procedures^2^, measurements were performed using the Multimode 5 Atomic Force Microscopy instrument (Veeco) upgraded to Multimode 8 version (Bruker). The images have been acquired in ScanAsyst mode.

**Electron backscatter diffraction (EBSD).** The surface sample of thin sections was water-free polished with lab prepared alumina suspensions (ethylene glycol based) of 1µm, 0.3 µm and 0.05 µm and finally polished with 0.05 µm colloidal silica suspension (water-free, Allied High Tech). Before analysis, samples were coated with a thin layer (*c.a.* 2 nm) of carbon using a high vacuum coater Leica EM ACE600. The EBSD study has was carried out with an Oxford Nordlys camera mounted on a Field Emission Scanning Electron Microscope (FE-SEM) JEOL JSM 6500F located in the Electron Microscopy Laboratory of the Spanish National Research Centre for Metallurgy (CENIM-CSIC). EBSD data were collected with CHANNEL 5 software at high vacuum, 10 kV, large probe current and 15 mm of working distance. EBSD patterns were collected at a resolution of 0.3 to 0.4 µm step size for crystallographic maps. EBSD data were processed using CHANNEL 5 from Oxford instruments. In this study, EBSD data are represented by crystallographic maps, band contrast images, pole figures and plot the ODF (orientation density functions), which represent the stereographic projection of crystallographic planes in reference to the (100) and (001) calcite planes. MATLAB^TM^ toolbox MTEX^3^ was used to calculate the correlated and uncorrelated misorientation.

**X-ray powder diffraction (XRD).** High-resolution powder X-ray diffraction measurements (PXRD) were carried out at high-resolution powder diffraction beamline (MCX) at the Elettra Synchrotron Radiation Facility, located in Trieste, Italy. The beamline is equipped with a Huber 4-axes X-ray diffractometer with a fast scintillator detector. Diffractograms were collected at room temperature for 5°≤ 2θ ≥ 55° with step: 0.01° at room temperature; the incident beam energy was set at 15 keV (0.08263 nm). Samples were contained in borosilicate capillary tubes with an inner diameter of 0.5 mm and rotated during measurements at a speed of 100 rpm. The structural parameters were refined by Rietveld analysis using the Fullprof software and a Pseudo-Voigt peak shape function^4^. The refined model was based on 16 structural parameters and 12 instrumental and background parameters.

**Electron microprobe analysis (EMPA).** It was conducted on polished slides with a carbon coating, with a JEOL Superprobe JZA-8900 with five wavelength-dispersive spectrometers, located at the National Centre of Electron Microscopy (the Universidad Complutense of Madrid, Spain). Nine elements (Ca, Mg, Sr, S, Ba, Na, Mn, Fe and P) were mapped at two gastroliths prisms with different patterns of alteration. The EMPA mapping enables simultaneous analysis of different elements and the generation of distribution maps for each element with 1μm resolution. An accelerating voltage of 20 kV with a beam current of 100 nA and a spot size and step interval of 1μm diameter (dwell time = 1000 ms) were used.


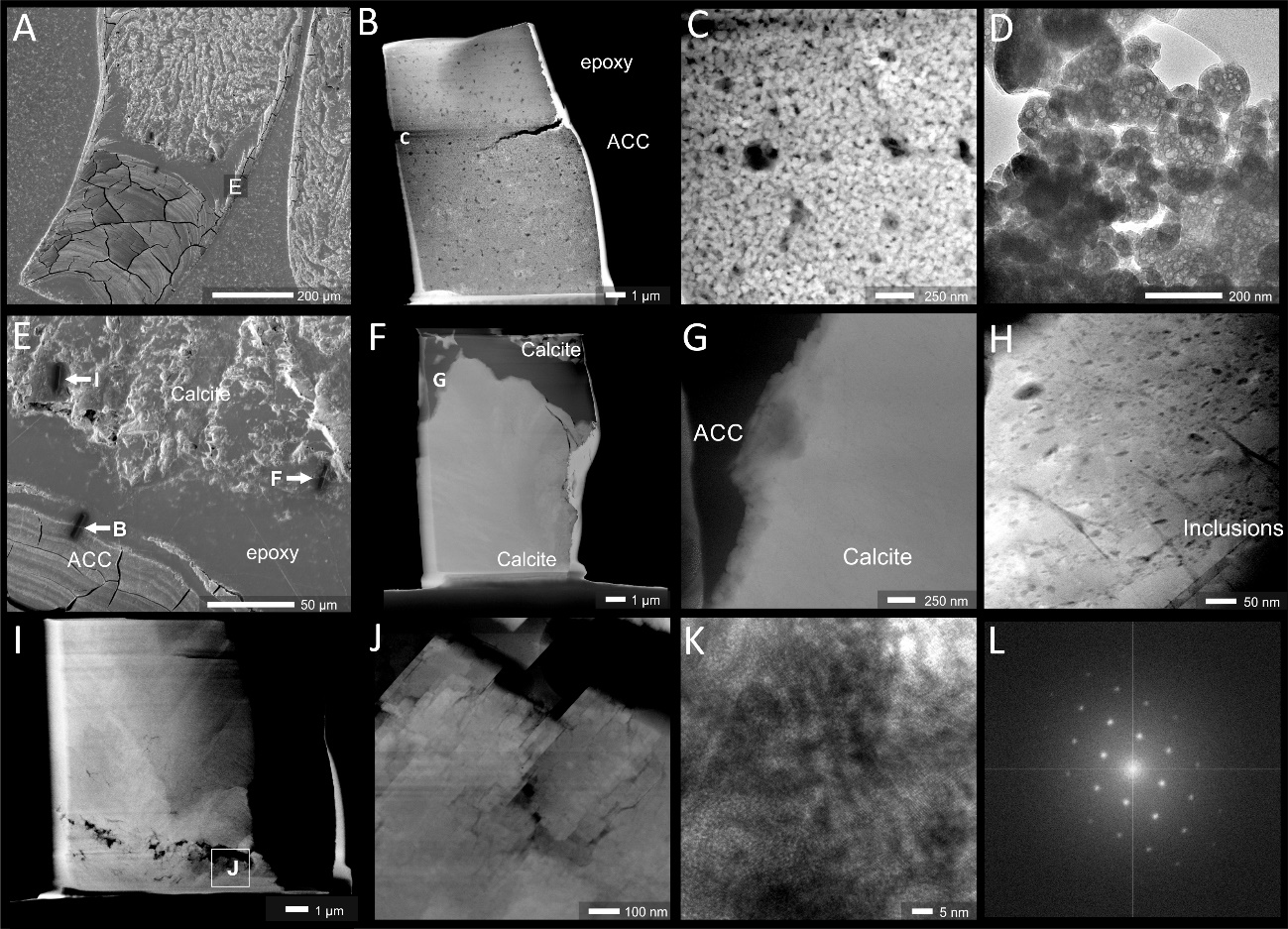


**Supplementary Figure 1. Transmission electron microscopy characteristics of pristine amorphous and secondary, crystallized regions of gastrolith columnar unit.** (A, E) Regions sampled by focused ion beam (FIB): pristine amorphous region (B-D), secondary calcite with still detected amorphous regions (F-H), secondary calcite (I-L). FIB lamellae shown in B, F, I. Amorphous region (B-D) shows well delineated round ca. 100 nm grains (C, D) that under higher magnification show some circular structures. In both calcitic regions (H, K) linear arrangement of nanometer-scale organic inclusions is visible. (J-L) High-resolution transmission electron microscopy (HR-TEM) image (K) of calcitic rhombohedral crystals (J) very well crystalline (L, fast Fourier transform analyses). ZPAL V.31/14.


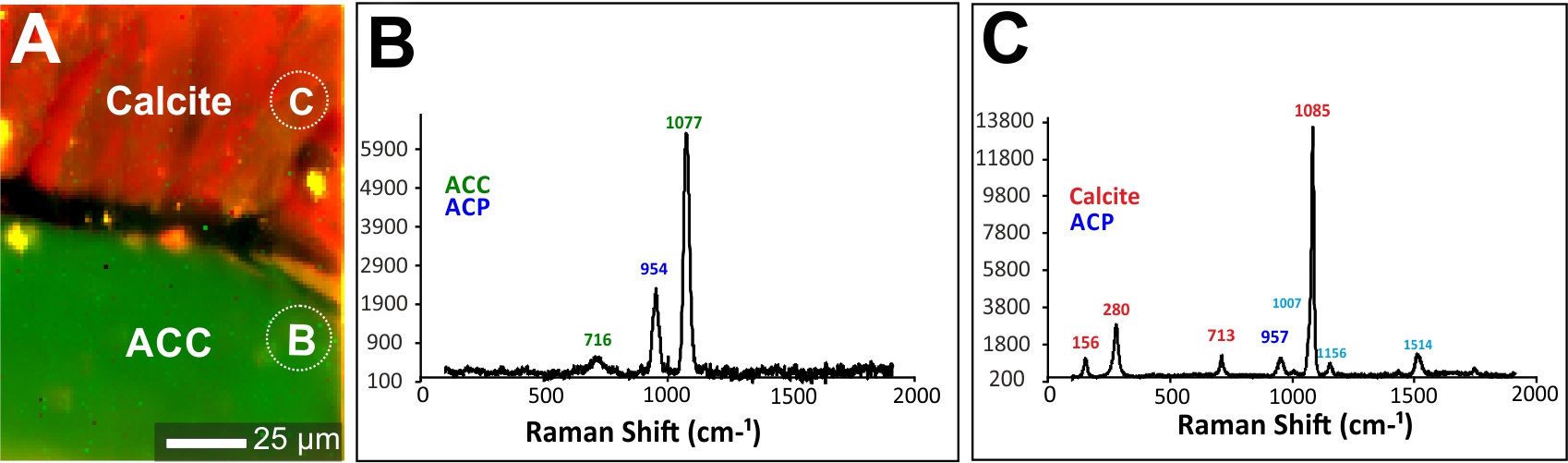


**Supplementary Figure 2. Micro-Raman spectra collected in the banded pristine gastrolith region and in the biomorphic calcite.** Approximate position of analyzed spots marked in A (as B and C circles). Note the presence of amorphous calcium carbonate (ACC), amorphous calcium phosphate (ACP), and calcite. ZPAL V.31/14.

**
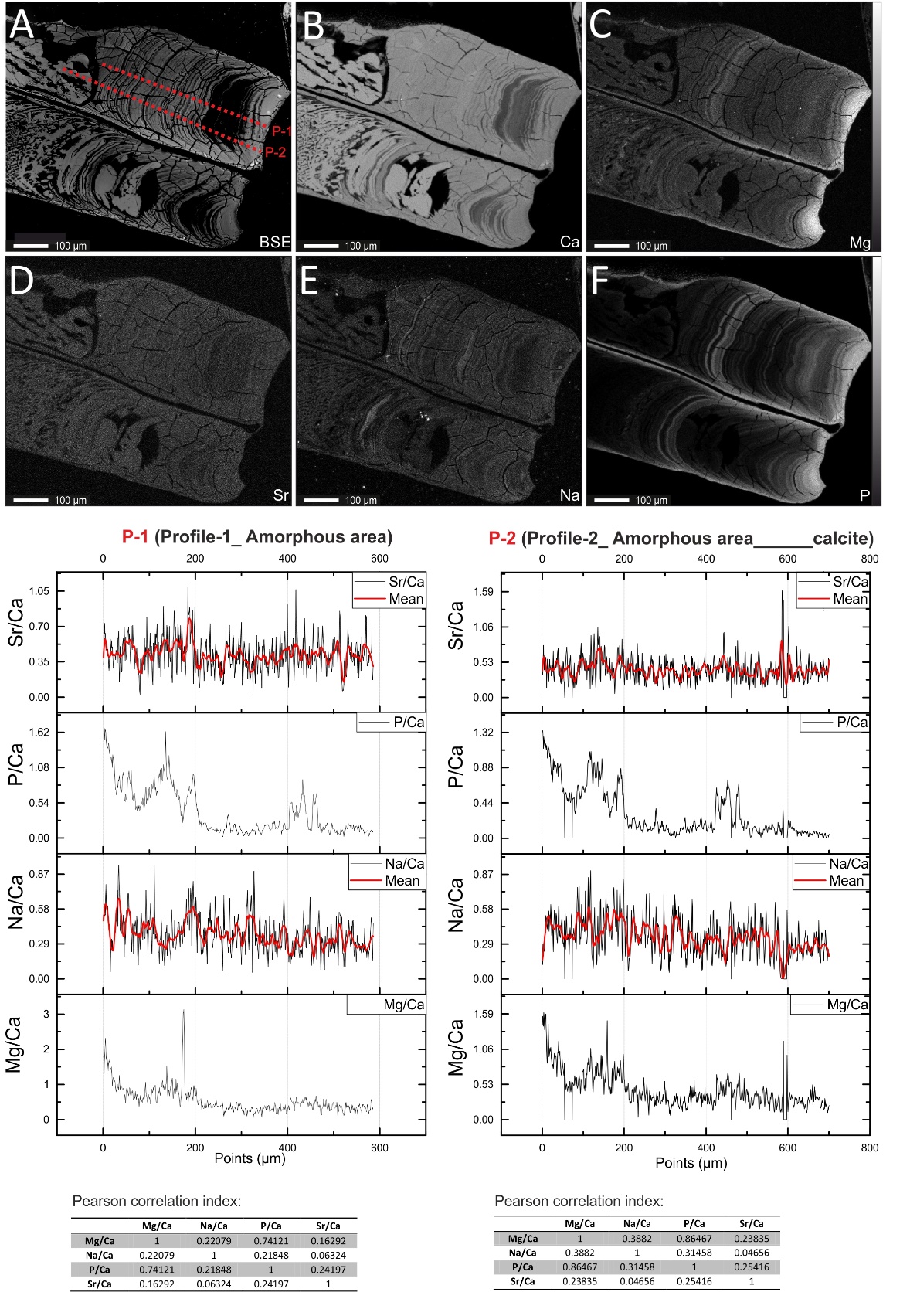
**

**Supplementary Figure 3. Electron Microprobe analyses (EMPA) of pristine amorphous and secondary, crystallized regions of gastrolith columnar unit.** Secondary electron image (A), BSE image (B), electron microprobe maps of Ca, Mg, Sr, Na, P (C-F, respectively), and transverse profiles (P-1; P-2). Metal/Ca ratio profiles (Sr/Ca, P/Ca, Na/Ca and Mg/Ca), P-1 and P-2. Red lines represent the simple moving average (SMA). Tables below summarize the Pearson correlation index between each metal/Ca ratio. Note the high co-varying between the P/Ca and Mg/Ca in each profile (0.74 and 0.86). ZPAL V.31/14.


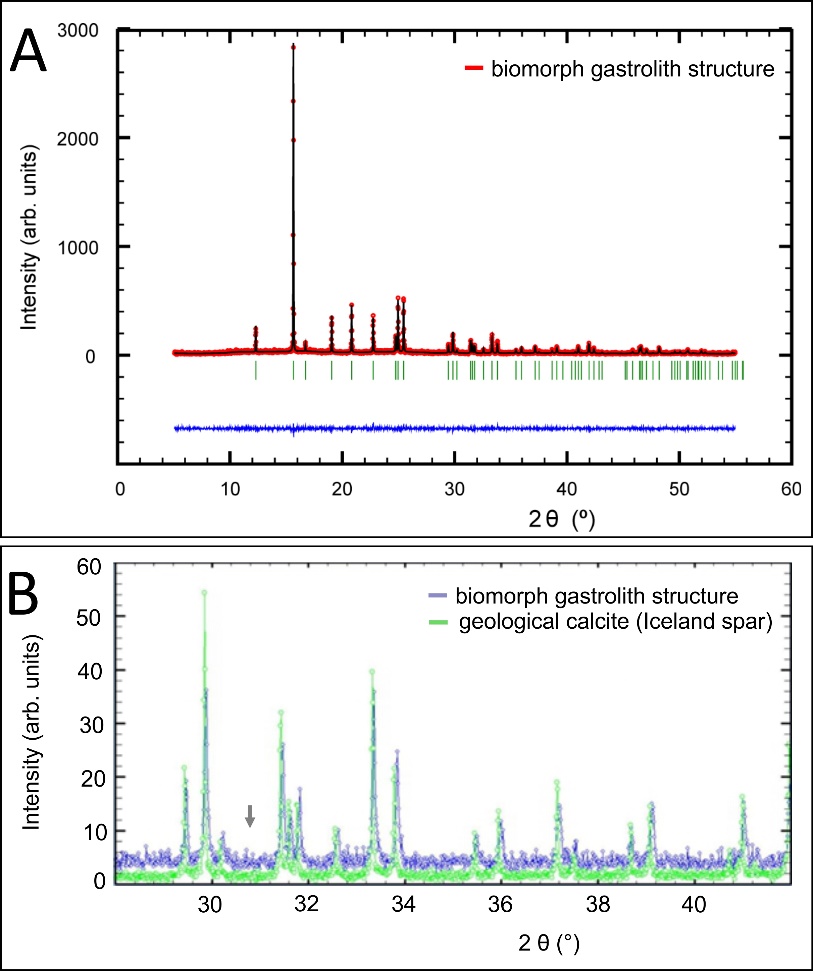


**Supplementary Figure 4.** Representative Rietveld plot for high-resolution PXRD of biomorph structures resulting from transformation of pristine gastrolith of *H. americanus*. (A) Continuous black line shows the measured data; red circles are the profile calculated by assuming the reference calcite structure. The blue line below the pattern shows the difference between the measured and the calculated patterns. The lower ticks indicate the positions of the Bragg peaks for calcite phase. (B) Enlargement of the Rietveld plot to show the lack of peak at around 31.8° (arrow) corresponding to the (211) crystal plane of hydroxyapatite. ZPAL V.31/14.


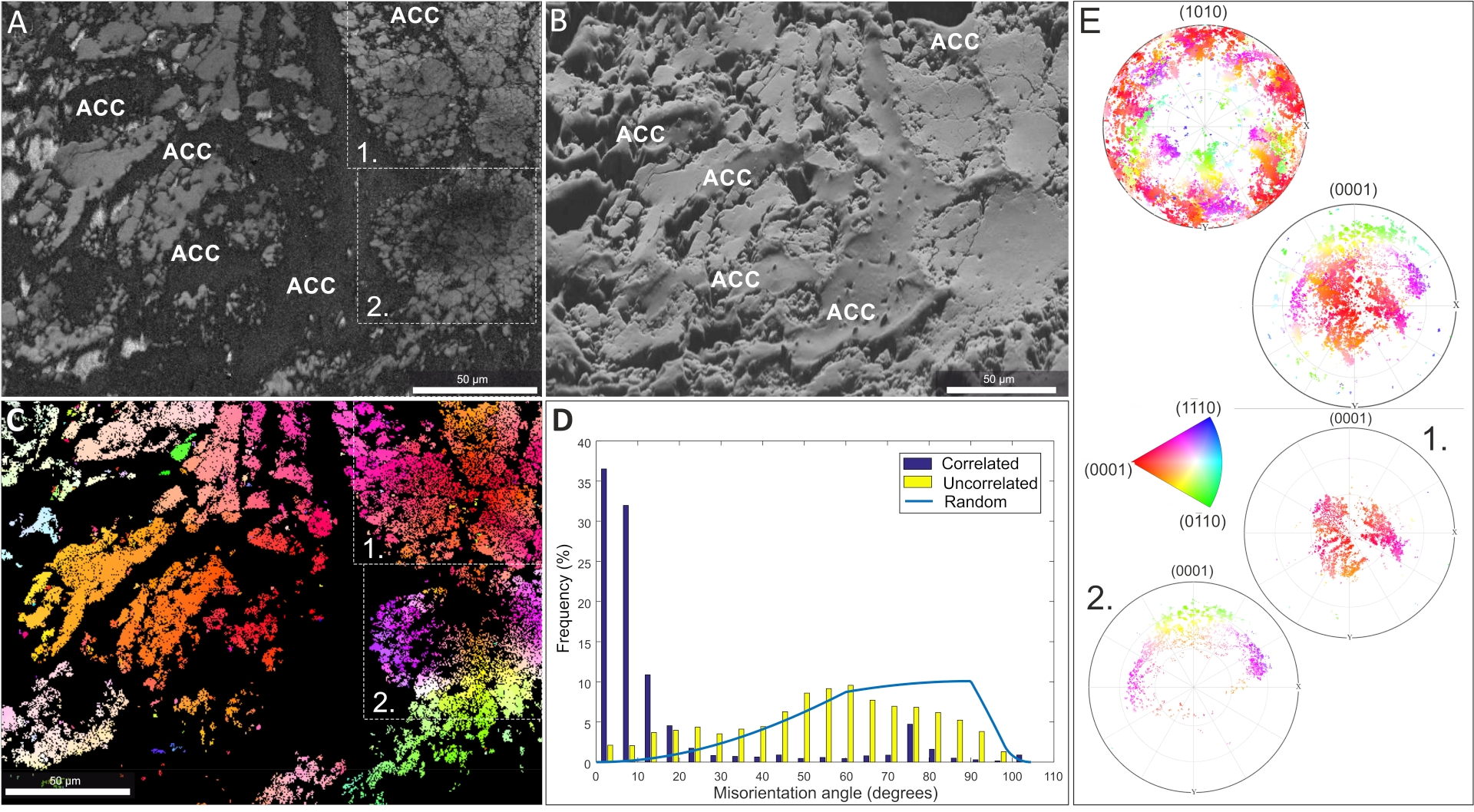


**Supplementary Figure 5. Crystallographic features of the transition zone between pristine amorphous phases and secondary biomorphic calcite with braided structures in upper part of gastrolith columnar unit.** (A) Band contrast images of the braided-like structure in an oblique section. Clusters of calcite crystals are embedded in amorphous material (ACC). Two spherulitic clusters belonging to transversal section of braided-like structures (regions 1 and 2 marked in A, B), both with three branches. Spherulitic clusters consist of smaller subunits. (B) SEM image of the area analyzed by EBSD. (C) EBSD orientation maps showing the crystallographic orientation of the studied area. (D) Histogram representing correlated (blue bars) and uncorrelated (yellow bars) misorientation of crystals (thick blue line represents random distribution computed for this crystal symmetry). Correlated misorientation has very low angles, showing highly crystallographic control of each unit, in contrast to a calculated uncorrelated misorientation distribution that exhibits systematically larger angles. (E) Pole figures (in reference direction view (x0) to the sample surface in a three-axis reference system) indicating the crystallographic orientation of calcite crystals with reference to the planes (10$\overline{1}$0) and (0001) and crystallographic key indicating color coding of crystallographic axes. The pole figures of areas 1. and 2. were plotted separately and show orientation of three branches that form each spherule. (F-I) Electron backscattered patterns (EBSP) of selected areas of panel (B). (F) background, (G) ACC, (H) partially disordered calcite crystals, with moderate electron diffraction and blurred Kikuchi bands, and (i) calcite crystal with well outlined Kikuchi bands. ZPAL V.31/14.


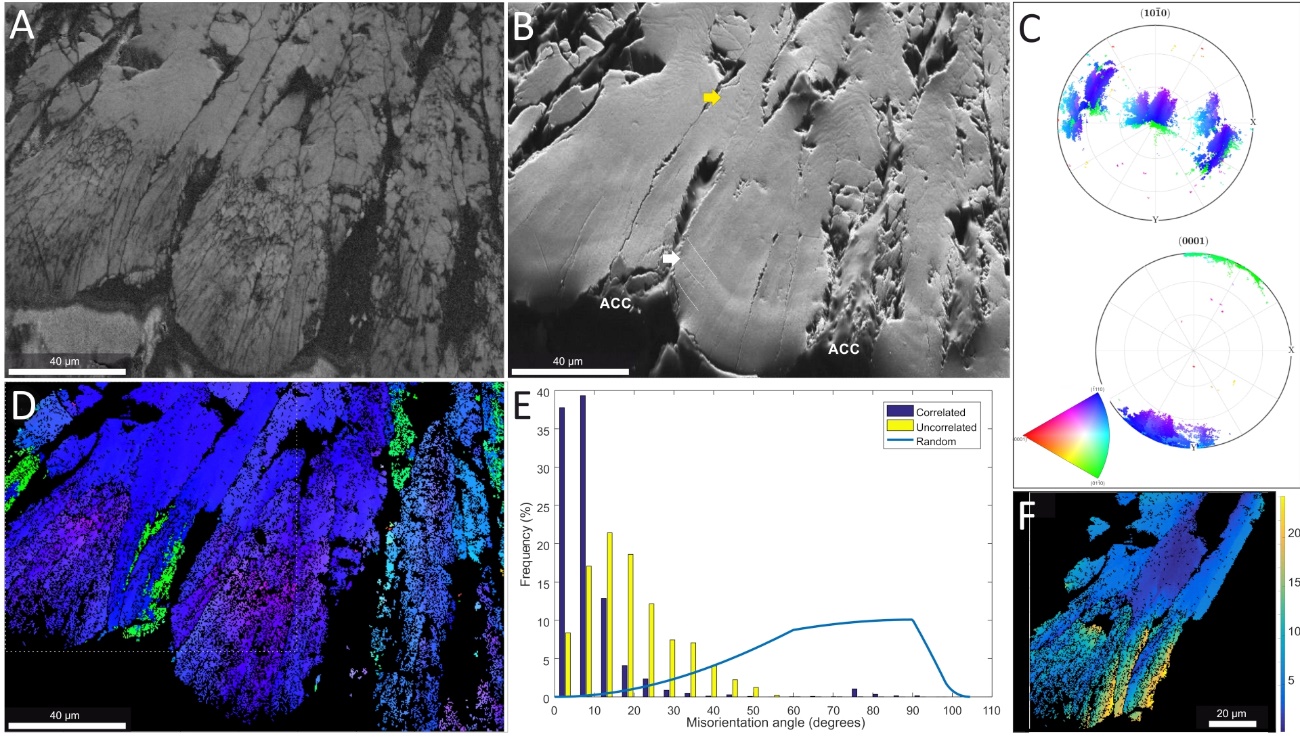


**Supplementary Figure 6. Crystallographic features of secondary biomorphic calcite with some remnants of original amorphous carbonate at growth front of braided-like structure.** (A) Band contrast (BC) image of braided-like structures, showing lobes at growth front; the crystal size decreases towards the edge of the lobes. (B) SEM image of the same area showing the arrangement of presence of amorphous carbonate at the edges of lobes and between them (note high solubility of amorphous regions during polishing). White arrow points to some growth bands inside the lobes and yellow arrow points small terraces of growth. (C) Pole figures (in reference direction view (x_0_) to the sample surface in a three axes reference system) indicating the crystallographic orientation of calcite crystals with reference to the planes (10$\overline{1}$0) and (0001) and crystallographic key indicating color coding of crystallographic axes. All the orientations are distributed in one pole maxima in the plane (0001) and three in the (10$\overline{1}$0), characteristics of calcite symmetry. Subtle disorientations of the crystals are located at the edge and within lobes (on regard to the main lobes), which show a straight variation in orientation. The pole maxima in the plane (0001) indicate that calcite crystals are oriented parallel to growth direction of gastrolith prism. (D) EBSD orientation maps showing the crystallographic orientation of lobes; note the homogeneous crystallographic orientation except in small areas at the edge and within lobes. Dashed square is showing the area analyzed in F. (E) Histogram showing correlated (blue bars) and uncorrelated (yellow bars) misorientation of crystals (thick blue line represents random distribution computed for this crystal symmetry). The correlated and uncorrelated misorientations have very low angles, showing highly crystallographic control of all units. (F) Misorientation image (vertical scale in degrees) of one lobe with same crystallographic orientation showing that the lobe is composed by a cluster of units (probably fibers as is shown in a). Fibers are most visible at the end of lobes. The crystallographic orientation inside the lobe varies forming bundles (herringbone-like), being most disoriented at the edge of lobes. ZPAL V.31/14.


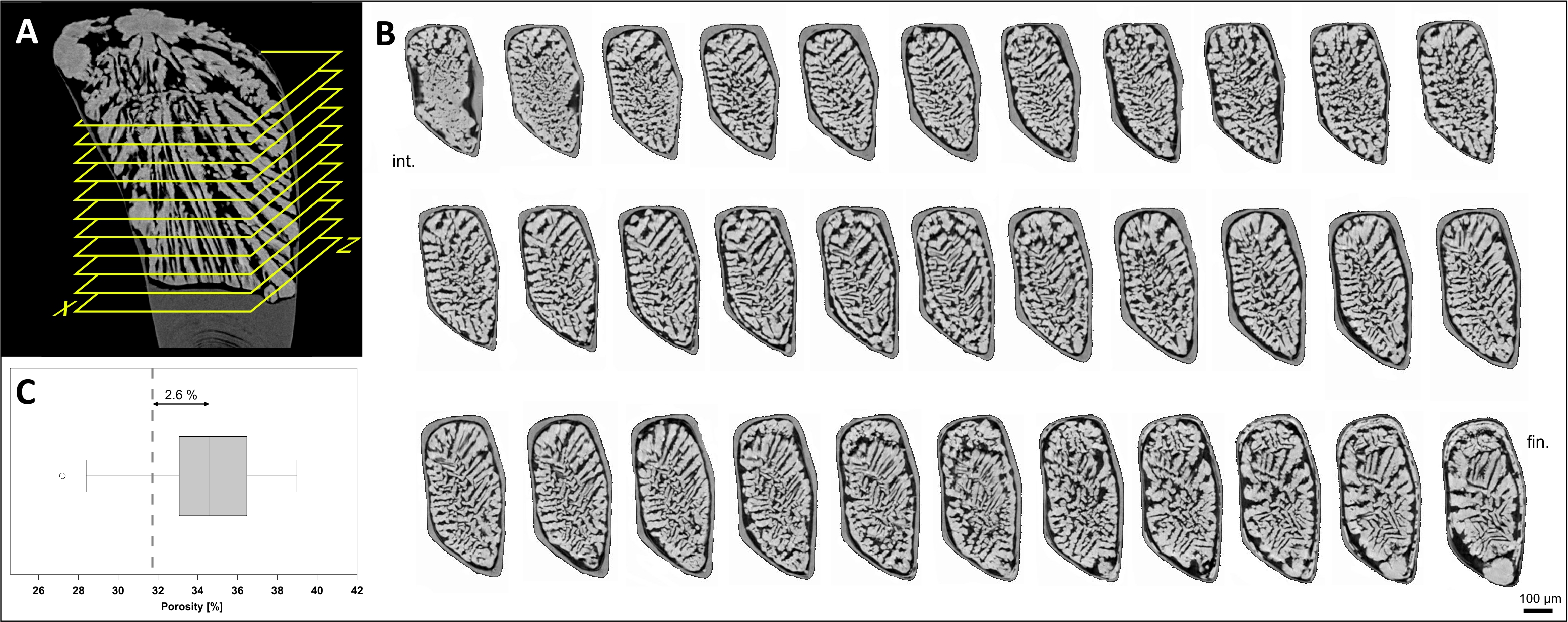


**Supplementary Figure 7. Calculation of porosity based on micro-CT images.** (A) X-ray image showing a longitudinal section of a columnar unit of gastrolith. Yellow squares represent the 30 sections taken in the planes X-Z and represented in (B). (B) 30 sections analyzed by ImageJ for the calculation of porosity in 2D. (C) Box chart showing the results of porosity calculation. Dashed line points the theoretical porosity formed by the direct transformation between ACC to calcite, considering the reduction in molar volume. int: initial section; fin: final section. ZPAL V.31/14.

| SAMPLE | χ2 | a | c | | volume | | Δa/a | Δb/b | Δc/c | | ΔV/V | | Crystallite size | | Microstrain | |
| --- | --- | --- | --- | --- | --- | --- | --- | --- | --- | --- | --- | --- | --- | --- | --- | --- |
| Units |  | [Å] | | [Å^3^] | | **[%]** | | | | **[%]** | | **[nm]** | | **[%]** | |  |
| Synthetic | 1.11 | 4.987385(16) | 17.05549(8) | | 367.401(2) | | - | - | - | | - | | - | | - | |
| *Pecten* | 1.17 | 4.99578(13) | 17.1008(6) | | 369.618(19) | | 0.168224 | 0.168224 | 0.265662 | | 0.603359 | | 209.69 ± 46.30 | | 0.019398± 0.017 | |
| Gastrolith | 1.07 | 4.99195(10) | 17.0528(5) | | 368.016(15) | | 0.091431 | 0.091431 | -0.01577 | | 0.167324 | | 312.25± 138.17 | | 0.056373±0.014 | |
| *Pinna* | 1.25 | 4.98269(8) | 17.0591(5) | | 366.788(13) | | -0.09424 | -0.09424 | 0.021166 | | -0.16692 | | 473.5496±239.68 | | 0.041714±0.012 | |
| Iceland  spar | 1.22 | 4.99595(9) | 17.0825(4) | | 369.248(13) | | 0.171633 | 0.171633 | 0.158365 | | 0.502652 | | - | | - | |

**Supplementary Table 1. Trigonal lattice parameters (Å) and unit cell volume (Å3) determined for calcite crystals of skeletons cultured under 3 pH (8.2, 7.6, 7.3).** Chi-square or goodness of Rietveld refinement (χ2). Macrostrain parameters (Δa/a; Δb/b; Δc/c) calculated in comparison with synthetic calcite (Fluka); microstructural parameters (crystallite size and microstrain) only were calculated for polycrystalline materials.

**References**

1. Schöne BR, Dunca E, Fiebig J, Pfeiffer M. Mutvei's solution: An ideal agent for resolving microgrowth structures of biogenic carbonates. *Palaeogeography, Palaeoclimatology, Palaeoecology* **228**, 149-166 (2005).

2. Stolarski J, Mazur M. Nanostructure of biogenic versus abiogenic calcium carbonate crystals. *Acta Palaeontologica Polonica* **50**, 847-865 (2005).

3. Bachmann F, Hielscher R, Schaeben H. Grain detection from 2d and 3d EBSD data--specification of the MTEX algorithm. *Ultramicroscopy* **111**, 1720-1733 (2011).

4. Rodríguez-Carvajal J. Recent advances in magnetic structure determination by neutron

powder diffraction. *Physica B: Condensed Matter* **192**, 55-69 (1993).
